# A qualitative study exploring perceptions and attitudes of community pharmacists about extended pharmacy services in Lahore, Pakistan

**DOI:** 10.1186/s12913-017-2442-6

**Published:** 2017-07-19

**Authors:** Furqan K. Hashmi, Mohamed Azmi Hassali, Adnan Khalid, Fahad Saleem, Hisham Aljadhey, Zaheer ud Din Babar, Mohammad Bashaar

**Affiliations:** 1 School of Pharmaceutical Sciences, Universiti Sains Malaysia, Penang, Malaysia; 2 Combined Military Hospital, Quetta, Pakistan; 3 Faculty of Pharmacy & Health Sciences, University of Baluchistan, Quetta, Pakistan; 4 College of Pharmacy, King Saud University, Riyadh, Saudi Arabia; 5 Department of Pharmacy, School of Applied Sciences, University of Huddersfield, Huddersfield, West Yorkshire, England; 6 SMART Afghan International Trainings & Consultancy, Kabul, Afghanistan

**Keywords:** Qualitative study, Perception, Attitudes, Community pharmacists, Extended pharmacy services, Pakistan

## Abstract

**Background:**

In recent decades, community pharmacies reported a change of business model, whereby a shift from traditional services to the provision of extended roles was observed. However, such delivery of extended pharmacy services (EPS) is reported from the developed world, and there is scarcity of information from the developing nations. Within this context, the present study was aimed to explore knowledge, perception and attitude of community pharmacists (CPs) about EPS and their readiness and acceptance for practice change in the city of Lahore, Pakistan.

**Methods:**

A qualitative approach was used to gain an in-depth knowledge of the issues. By using a semi-structured interview guide, 12 CPs practicing in the city of Lahore, Pakistan were conveniently selected. All interviews were audio-taped, transcribed verbatim, and were then analyzed for thematic contents by the standard content analysis framework.

**Results:**

Thematic content analysis yielded five major themes. (1) Familiarity with EPS, (2) current practice of EPS, (3) training needed to provide EPS, (4) acceptance of EPS and (5) barriers toward EPS. Majority of the CPs were unaware of EPS and only a handful had the concept of extended services. Although majority of our study respondents were unaware of pharmaceutical care, they were ready to accept practice change if provided with the required skills and training. Lack of personal knowledge, poor public awareness, inadequate physician-pharmacist collaboration and deprived salary structures were reported as barriers towards the provision of EPS at the practice settings.

**Conclusion:**

Although the study reported poor awareness towards EPS, the findings indicated a number of key themes that can be used in establishing the concept of EPS in Pakistan. Over all, CPs reported a positive attitude toward practice change provided to the support and facilitation of health and community based agencies in Pakistan.

**Electronic supplementary material:**

The online version of this article (doi:10.1186/s12913-017-2442-6) contains supplementary material, which is available to authorized users.

## Background

During the past few decades, pharmacy services witnessed a transition of roles of professional practice. The count-and-pour system replaced the customary compounding role, and more recently was transformed to the delivery of pharmaceutical care. Since the adoption of pharmaceutical care in the early 90s, the role of pharmacist changed from being product oriented to patient-focused model [1, 2]. Therefore, the practice of pharmaceutical care is applicable in all settings i.e. community, hospital, clinics and long-term care [3]. Within this context, pharmaceutical care has evolved to embrace different services at the community level. These services range from brief counselling following medication purchase to lengthy extensive counselling services and other value added services known as extended pharmacy services (EPS) [4].

Extended pharmacy services are referred to those services which are not associated with traditional services offered by the pharmacists such as dispensing and providing individual consultations on prescription and over-the-counter (OTC) medications, but include new series of services for example, medications therapy management (MTM), home medication review (HMR) which involve comprehensive medication reviews to look for medication-related problems and all aspects of chronic disease management (CDM) which may include screening, patient education and knowledge, disease monitoring and communication with the primary healthcare team [4, 5]. Extended Pharmacy Services require additional or special skills, knowledge and/or facilities, and are provided to people with special needs [6]. In addition to the provision of safe and effective prescribed pharmacy- and self-selected medicines, a growing diversity of additional services are being developed and remunerated in developed countries [6] and the new role of community pharmacists as a key personal of the multidisciplinary provision of healthcare is openly accepted [7]. In Australia for an instance, community pharmacist is considered as a health practitioner and educator for the public [8]. In the United Kingdom (UK), government has introduced New Medicine Service (NMS) for community pharmacists to promote self-care and to improve the management of long term conditions [9]. In the US, community pharmacist is considered as a readily accessible primary healthcare provider [10], while the community pharmacists in Canada play proactive role as providers of primary healthcare services [11, 12]. On the contrary, community pharmacy practice is totally different in the developing countries and the practices are typically confined to traditional practices of dispensing and a business oriented approach is followed [7, 13]. Shifting our concern to the concept of EPS in Pakistan, the notion is still naïve to the community pharmacists and public. Pakistan is the 6th most populous country in the world [14]. The healthcare system of the country is a three-tiered structure, with primary, secondary and tertiary healthcare units [15]. The sales of medicines, by way of retail and wholesale, are regulated by the Drug Act 1976 [16]. The practice of pharmacy, education, establishment of council (Pakistan Pharmacy Council and Provincial Councils) and registration pharmacists are under the provisions of Pharmacy Act 1967 [17]. The registration of graduate pharmacists is a straightforward process. The graduates from recognized institutions and universities providing pharmacy education, apply for the registration to the respective Provincial Pharmacy Councils, as pharmacist, ‘qualified person’ to be registered under, ‘Register A’, termed as ‘Category A’ (certificate of registration), under Section 23 of the Pharmacy Act 1967 [17]. The provincial council in the province of Punjab is known as the Punjab Pharmacy Council (PPC), situated in Lahore. All the study respondents in our study setting are legitimate registered graduate pharmacists with PPC, Lahore. According to the record of PPC 15,702 pharmacists have been registered as of December 2016 [18]. The qualified pharmacists (registered) apply for license to practice as community pharmacist in the Office of the Executive District Officer-Health (EDO-Health) Department of Health, City District Government, Lahore.

In contrast to the situation in developed countries, community pharmacists are underutilized as they are not considered as healthcare professionals and hence their role is neither identified nor recognized in the healthcare system [19]. This lack of recognition is attributed to limited interaction of pharmacists with people as well as lack of awareness among public [20, 21]. As far as community pharmacy services are concerned, like other developing countries, pharmacists are involved in the dispensing of medicines and follow a business-oriented approach. Prescription handling and patient’ management is least seen at community pharmacies in Pakistan [22, 23]. Although a small number of pharmacies offer patient counselling services, overall provision of community pharmacy services is poor in Pakistan [21]. In majority of the cases, pharmacies in Pakistan are owned by non-pharmacist (businessmen) and they are more focused toward flourishing their business rather than focusing on patient care and other patient-oriented services. There is a lack of sufficient pharmacists to work as well as the physical presence of pharmacists at the community pharmacies are the challenges faced currently in Pakistan. Generally, majority of community pharmacists are unaware of the concept of extended pharmacy services [24]. There is lack of awareness in public about the role of community pharmacist as well as the collaboration among the healthcare professionals, as pharmacist-physician interaction is not there in appreciable extent [25].

Therefore, this qualitative study was conducted with an objective to explore the perception and attitude of community pharmacists about their extended role in the community pharmacies in Lahore, Pakistan.

## Methods

### Study design

A qualitative methodology was adopted to explore the perception and attitude of community pharmacists about the EPS. We adopted a qualitative design for various reasons. The design is flexible and consents to an in-depth exploration of respondents’ attitudes, experiences and intentions [26, 27]. In addition, qualitative methods generate a wide range of ideas and opinions that individuals carry out about the issues, as well as divulge viewpoint differences among groups [27]. Furthermore, for under discovered research areas, qualitative methods attempt to fill in gaps that are left unexposed by survey based research [28]. Therefore, inline to the objectives of this study, qualitative interviews were a superlative choice for inductive approaches aimed at generating concepts and hypothesis that have far more potential for research than any other models [29].

### Instrument development

Based on an in-depth literature review [19, 30–49] and current prevailing practices of community pharmacy in Pakistan, a semi structured interview guide was developed [50]. The guide focused on knowledge of EPS, perception towards current community pharmacy practice in Pakistan, knowledge and confidence in practice change, customers’ feedback, and future interventions towards practice change. The guide was subjected to validation and reliability assessment prior to data collection. Qualitative experts at Universiti Sains Malaysia validated the interview guide by using a combination of argumentative and cumulative techniques. Argumentative validation uses data as a squabble source to overcome one contradictory viewpoint [51]. Cumulative validation is the method of cross referencing whereby the researchers use accessible literature to match the findings. Based upon the nature of data and availability of resources, the present study followed cumulative process of validation. Reliability of research was assured by preserving records of face-to-face interviews of the respondents of the study. The guide was piloted on two community pharmacists and was modified accordingly. As mutual consent of the experts and CPs was received, the interview guide was made available for real-time study (refer to Additional file 1).

### Study setting

The study was conducted in Lahore. It is the capital of the province of Punjab, second largest and most populous city of Pakistan having a little over 7.2 million inhabitants with 81.17% urban settlement [52]. Beside the reasons of being the capital city and second most populous, Lahore is considered as the major centre for health facilities, education, business and culture. The community pharmacy sector is considered to be well established in Lahore, having both independent- and chain-type of pharmacies.

### Respondents and inclusion criteria

There are 3618 registered licence pharmacies in Lahore [53]. Community pharmacists, registered at the Punjab Pharmacy Council (PPC) and practicing either at independent and chain pharmacies with minimum 8 h per day presence at pharmacy were targeted for data collection. Moreover, only those pharmacies were targeted which employed full-time licensed pharmacists. Pharmacy technicians and non-registered pharmacists were excluded from the study.

### Sampling, data collection and processing

Participants were recruited using purposive methods until saturation of themes was achieved. Purposive sampling was done based on the preconceived ideas about the required characteristics of the sample. Thus, targeting and identifying those individuals who had experience in community pharmacies and were influential about service development. Interviews with consented participants were conducted after individual appointments. The interviews were conducted in English (pharmacists, generally are good in English speaking as the medium of instruction in institutions and universities is Pakistan is exclusively in English language). Each interview lasted for 30 to 40 min. Probing questions were asked and participants were given freedom to express additional views and comments. All interviews were audio-recorded and the principle researcher took additional field notes. The saturation was achieved at the 10th interview; however, two interviews were carried out further to confirm the saturation. The interviews were verified for accuracy and consistency by listening to the recordings. The first author analysed the transcripts line by line, which were read repeatedly and thematically analysed for their contents. Co-authors of the study verified the emerging themes and contents.

## Results

Twelve CPs were interviewed. All participants were males (as females do not prefer to work in community pharmacies due to social reservations). Majority (*n* = 8) of the respondents were from the age group of 20–30 years and six were the managers of the community pharmacy. Eight CPs had a working experience of 1–5 years at community pharmacies as shown in Table 1.

**Table 1:** Demographic characteristics of the study respondents (*n* = 12)

Characteristics	Frequency
*Age (years)*	
20–30	8
31–40	1
> 40	3
*Gender*	
Male	12
*Educational status*	
B. Pharmacy	5
Pharm. D	2
M. Phil	5
*Experience (years)*	
1–5 years	8
6–10 years	1
> 10 years	3
*Status in Pharmacy*	
Employee	5
Manager	6
Proprietor	1
*Prescriptions filled/day*	
1–20	2
> 20	10
*Location of pharmacies*	
Urban	12

During the analysis, five major themes were identified; (1) Familiarity with EPS, (2) current practice of EPS, (3) training needed to provide EPS, (4) acceptance of EPS and (5) barriers toward EPS.

### Theme 1: Familiarity with the extended pharmacy services (EPS)

During the interviews, the respondents were asked for their knowledge and information about EPS. Majority of the respondents had no idea about the EPS while a few CPs reported a little familiarity with EPS. Only two CPs had updated knowledge about EPS. This may be attributed to the fact that the young pharmacists with experience of 1 to 2 years keep themselves updated with the latest developments taking place in pharmacy practice around the world.

*“No I don’t have any idea about extended pharmacy service, its new to me however we are offer services as blood pressure monitoring, blood sugar monitoring, counselling, but more confined to BP and sugar monitoring services only” (CP4)*

*“I have slight idea about extended pharmacy services. We also give services like BP monitoring, blood sugar test, body weight and advice about the medicines patients are using and we also address issues regarding prescriptions” (CP3)*

The pharmacist having good update knowledge about the extended pharmacy services expressed views as:

*“The role of pharmacists beyond the typical dispensing services is known as the extended pharmacy services and health care services, I conduct MTM service on my own” (CP1)*

### Theme 2: Current system of practice and EPS in community pharmacies in Lahore

The overall response of participants regarding the current system of practices and sales in community pharmacies was uniform. Community pharmacies typically work as a shop with no concept of keeping patient specific information as patient history, allergies or medical record. Provision of EPS at the community pharmacies was least reported and principal focus of community pharmacy was increased sales through medicines and other products.

*“The system of practice is typically dispensing, patients present their prescription at the counter the technician or pharmacist simply handover the medicine and charge” (CP7)*

“*Community pharmacy practice is typically selling of medicines in Pakistan it is not well defined and far from the concept of EPS” (CP8)*

### Theme 3: Training needed to provide EPS

Training of pharmacists was considered an important component for better services. Majority of the CPS had no formal training before joining the community pharmacy practice. The employer provided training for a certain period while working in the pharmacy. The CPs responded in a positive way to this aspect of pharmacist’s capacity building. Respondents also added that for provision of EPS, they need training so that they can collaborate with other stakeholders at the community level.

*“The training of pharmacists regarding extended pharmacy services is very important and it must be under the supervision of the government and organized training programs should be there” (CP2)*

*“Continuous education programs for community pharmacists are necessary and should be conducted on regular basis by the pharmacy council” (CP6)*

### Theme 4: Acceptance of EPS

Majority of the CPs were positive towards EPS and ready to accept the change. However, CPs had the reservation that the government must take initiative so that they can provide EPS without any apprehension and uncertainty.

*“Definitely making good standards of practice, laws and regulation with emphasis on EPS we will be able to be at par with any place in the world”(CP11)*

*“Yes it’s necessary in Pakistan where a significant number people living cannot afford to visit physicians, therefore for easy access to quality medicines pharmacist can play extended role in public health”(CP9)*

### Theme 5: Barriers towards adoption of EPS

Community pharmacists were asked about the barriers toward adoption of EPS in Pakistan. Lack of training programs for CP, amendments in existing laws and prevailing government policies were considered as barriers to practice change. Other limitations indentified by the participants were poor salary structures, insecurities in job, shortage of pharmacist and renting out of pharmacists’ practicing licenses.

*“Well the barriers are……….the law doesn’t clearly state what pharmacists are allowed to do? Educating the people or creating awareness about the pharmacist and its professional role. Developing services in a way what people want to be treated like” (CP1)*

*“The government should provide the job description for community pharmacists as there is no such binding…EPS need to be indentify to start with by the government ……on the part of pharmacies we can help develop the human resource and can help in building the capacity of community pharmacists” (CP4)*

In addition to that patients’ acceptability of advice from pharmacist, the role of physicians was also pointed out as a barrier.

*“There are barriers as patient has not much confidence on pharmacist…..after counselling they still prefer to consult their physician …Or in other words our healthcare system is dominated by doctors……only small percentage of patients accepts the pharmacist’s intervention. Another major barrier is the timing or the patients are always in hurry, it’s just a shop of medicines for them” (CP6)*

## Discussion

As far as community pharmacy services and practices are concerned, EPS is a new concept in Pakistan. Within this context, although several studies are reported in literature on the delivery of EPS in developing countries [54–56], however there is paucity of information from Pakistan and knowledge and perception of CPs towards EPS is uncertain. The present study reported poor familiarity of CPs towards EPS in Pakistan and suggested multiple factors that must be addressed to bring practice change at community pharmacies in Pakistan.

A handful of our respondents had a clear idea and concept of EPS; however, majority of the respondents were unaware of EPS. This warrants insufficient exposure of pharmacists during their undergraduate studies as pharmacy curriculum in Pakistan follows traditional classroom teaching methodology. In fact, the pharmacists are required to have knowledge and skills extending beyond the typical roles in the community pharmacy [57]. In addition to the lack of exposure, practicing pharmacists in Pakistan often fail to update their skills and knowledge as per trends and innovations being used and introduced around the globe. Therefore, in order to maximize the utilization of CPs at the community level, a set of unique structured strategies and health system reforms are required to be introduced to enhance the knowledge and working capacity of CPs in Pakistan [58]. These strategies must aim to introduce trainings for CPs that can help in increasing the confidence in providing EPS to the population. These trainings on patient-centred activities should be on regular basis in the form of continuous pharmacy education (CPE) and continuous professional development (CPD) programs. These programs must be accredited from the Pharmacy Council. The CPE may include the aspects of EPS such as; MTM, HMR, CDM and information about public health issues. Additionally, the pharmacy curriculum should be designed to have aspects of patient care to be practiced by CPs and render services to public. A confident and well-trained pharmacist will be able to offer public health services more actively and will likely to have a positive impact on health-related issues [59]. The government should organize educational programs for community pharmacists to equip them for their extended roles in community practice [60].

The current scenario of community pharmacy practice in Lahore can be explained by the Transtheoretical Model of change (TTM) [61]. Transtheoretical model explains the behaviour change and the readiness of an individual to act on new behaviour, provides new strategies or processes of change and guides individual through different stages if change and sustenance of the change. Since, the present study intended to explore the current trends of practices, readiness of CPs towards practice change and provision of EPS, therefore, we believe this behaviour may best be explained by the TTM. Based on the model, CPs working in Lahore had a mixed behaviour towards need for practice change. The study has shown that those having more than 10 years of experience (aged 31 to >40) were in pre-contemplation stage or had no clear idea about the EPS and pharmaceutical care. On the contrary, young pharmacists with experience ranging from 1 to 5 years (aged 20 to 30) seem to be at the contemplation stage. The results notwithstanding, it is important that the pharmacists should be educated properly about their extended roles while working in community pharmacy during their undergraduate studies. It should also be addressed by the government to provide on job certified trainings (accredited CPE programs) to pharmacists and formalize the provision of EPS. This will create awareness among CPs and can shape as a mediating factor in the development of the preparation phase. Once the CPs join practice, continuous monitoring through the respective stakeholders such as; health department, pharmacy council and professional pharmacy association, addressing the healthcare needs of public will result in the provision of EPS (action) and maintenance.

Our study findings are supported by the work done by Menghuan Song and co-workers, in China, stating that poor familiarity of community pharmacists towards EPS is linked to limited care knowledge and lack of human- and financial resources [62]. The current study revealed that only some aspects of pharmaceutical care are conducted by CPs in community pharmacies and the CPs are conducting services on their own, for instance following up patients with chronic diseases as diabetes and hypertension. Similar sort of activities have been reported in community pharmacies of Khartoum, Sudan [63]. The advice to patient particularly about the lifestyle modifications is considered to be an important part of CP’s job, however, only few CPs were ready and able to provide such services and they were of the opinion that some extra remuneration would be helpful in addition to their salary package being offered by the pharmacy they work for [64].

Despite the easy access of public to pharmacies and CPs, there is still lack of recognition of pharmacists as healthcare provider by other stakeholders (physicians, nurses, paramedics) of healthcare system in Pakistan. This finding of study is in line with those studies carried out in majority of the developing countries [22, 65]. Sales of drugs as well as prescription only medicine (PoM), even antibiotics are being dispensed or sold without prescription is a common practice in majority of the pharmacies in the city [66]. The overall process of prescription handling, history taking and medication counselling is either limited, only few pharmacies offer, or seldom present in majority of pharmacies [22, 65]. Provision of proper records of patients in integrated manner should be maintained and made mandatory by the government at each community pharmacy to serve the society in a better way [25].

Pharmacists working in community pharmacies in Pakistan are not trained for their role in rendering EPS. The community pharmacists usually work in isolation from the rest of the healthcare team, predominantly working as drug retailers and drug dispensers. Pharmacists are believed to be well suited to assume an extended role in the healthcare system, despite their current role as retailers [67]. Taking on as patient-focused responsibilities is commensurate with the profession’s training and expertise. The CPs working in the current study setting are pharmacy graduates of two streams; an old scheme of study known as bachelor of pharmacy (B. Pharmacy) with 4-years duration, while the other introduced in 2005 as undergraduate study 5-years duration known as the doctor of pharmacy (Pharm. D), it was designed to have clinical contents with more patient oriented approach [68]. Despite of this scheme of study the pharmacists are still lacking in the trainings required for the practical field. The Pharmacy Council of Pakistan and the provincial councils should take initiative for the training of community pharmacists for their extended roles. The government should introduce such schemes that people would know pharmacists’ role as primary healthcare workers provided pharmacists are appropriately trained. Preparing and teaching current pharmacy students to be resourceful for their extended roles in community pharmacies will be beneficial for profession and the healthcare system as a whole [5].

The respondents of study expressed their views about the readiness and acceptance of change. It is important that CPs in the study setting be prepared for the acceptance and changes in the practice to provide quality patient-centred services. The transformation or reform can be difficult and challenging even in the developed countries [69]. Recently, developed countries as Australia, Canada, England and the USA have undertaken a range of reforms with the objective of equipping CPs with extended roles and responsibilities when caring for people with multiple chronic conditions however, the pharmacists found it to be challenging to accept this change [4, 38, 69]. The government needs to play proactive role to make laws to establish EPS in community pharmacies. There should be a liaison between the pharmacists and other healthcare workers.

There were several barriers to effective pharmacy practice model identified that are perceived by the CPs working at community pharmacies. Our study reports lack of sufficient human resource as community pharmacists to cater for the needs of huge population as one of the major barriers [26]. The common practice in Lahore is that pharmacists usually rent their licenses to the owners of pharmacies without being physically present on the pharmacy premises. This can be considered as another important barrier. Recently, the Drug Court, Lahore has ordered the drug inspectors (regulators) all over Punjab to ensure the physical presence of pharmacists at the pharmacies otherwise they could cancel the license and impose penalties as well. Public awareness about pharmacists has been perceived as another barrier. Despite of 80% urban population of our study setting, people still have very little idea about the services that a pharmacist could offer them for their health-related issues. There is a cycle of dysfunction in which pharmacists hindered by time do not provide patient care beyond dispensing of a product, as a result patients rely on their physician for advice about medications and they do not acknowledge the pharmacist as an advisor for medication [70]. This sort of behaviour is believed to be responsible for creating no motivation for both patients and CPs. This can be overcome by creating awareness about the pharmacist’s healthcare role in the public. Similar findings were presented by Xiang Lang and co-workers in Pakistani healthcare system stating that there is a gap between the public and the pharmacist, which can only be plugged in by creating awareness among public regarding pharmacists’ role in healthcare system [71]. Even in developed countries the patients or public have lack of trust on the care services provided by the community pharmacists [72]. The public’s trust can be developed by increasing the quantity of patient-pharmacist interactions and quality of service [43] and gaining GP support of EPS [73, 74]. Another important barrier identified was the salary structure of CPs working in community pharmacies. Currently the remuneration and salaries are inappropriate that lead to the discouraging working environment for pharmacists. In fact, this is one of the reasons of less number of pharmacists making community pharmacy a career. Adequate remunerations are believed to be necessary for provision of extended pharmacy services warranting better public health [75].

The role of pharmacists in patient care emphasized, as one of the respondents, mentioned that the affordability medicines and access to physician was compromised for a significant number of people in Pakistan. Addressing provider-related barriers could help in better access to healthcare services [76]. In this scenario, CPs, could be a good choice to help people for the treatment and advice for their minor illnesses through community pharmacies.

### Limitations of the study

This study was conducted in Lahore which is the second largest city in Pakistan. The results of our study may not be representing the trends of EPS in the whole country.

## Conclusion

This study showed a lack of awareness of majority of CPs about the EPS and pharmaceutical care in community pharmacies in Lahore, Pakistan. However, the CPs showed a good attitude toward practice change provided the government plays its role to remove the barriers for the implementation of EPS in community pharmacies and ultimate benefit of the public for primary care issues.

## Additional file

Additional file 1: The file entitled ‘interview guide’ is the semi structured interview guide. The file contains the questions in sequence that were asked during the interview sessions. (DOCX 18 kb)

## Abbreviations

CDM: Chronic disease management
CPD: Continuous professional development
EPS: Extended pharmacy services
HMR: Home medication review
MTM: Medications therapy management
NMS: New medicine service
OTC: Over-the-counter
PoM: Prescription only medicine
PPC: Punjab pharmacy council
