# Supplementary material for: A qualitative study exploring perceptions and attitudes of community pharmacists about extended pharmacy services in Lahore, Pakistan
Source: BMC Health Serv Res. 2017 Jul 19;17:500. doi: 10.1186/s12913-017-2442-6 (PMC5518160; doi:10.1186/s12913-017-2442-6)
Supplement: Additional file 1: — The file entitled ‘interview guide’ is the semi structured interview guide. The file contains the questions in sequence that were asked during the interview sessions. (DOCX 18 kb) [file 12913_2017_2442_MOESM1_ESM.docx]

**QUALITATIVE PHASE**

**COMMUNITY PHARMACISTS’ INTERVIEW GUIDE**

**Objective:**

Exploring the perception of community pharmacists towards the extended community pharmacy services

**Part I**

***Focus: Current community pharmacy practice***

1. Do you have any idea about **extended pharmacy services**?
2. Have you **ever conducted any healthcare service**? **How frequently** you conduct health care services?
3. Do you feel **your role has changed** when offering these services – and how this **makes you feel** (increased job satisfaction, helping customers, increased job load)?
4. **How long the average service** / conversation taking? Do you have enough time and how has this impacted on workload?
5. **Who is providing the services** – yourself or pharmacy staff (trained technician) etc.?
6. Do you have **trained technicians** or they are trained on experience basis?
7. Have you **briefed** the rest of the pharmacy team to **refer to the pharmacist**?
8. What is the **feedback** from the rest of your team about medicines related information?
9. Any **recent sample of the service** being offered to customers (prompt for conversation, symptoms, recommendations / advice given)
10. What is your **opinion regarding the current system** for medicines sales by community pharmacists in Pakistan?
11. Is there any **recording system for the patient information**? If system is present, what difficulties are faced?
12. Any idea about the **difference of community pharmacy practices** between Pakistan and other developed countries?
13. In your opinion, do you think that there is a need **for community pharmacy practice-change** in Pakistan?

**Part II**

***Focus: Knowledge and confidence towards community pharmacy practice-change***

1. What is your understanding **pharmaceutical care concept** and provisions?
2. Do you have the **confidence in approaching customers** for health care intervention? Are there any limitations or barriers?
3. Do you think you have the **ability to deliver an extended role** of pharmacists? What, if anything, needs to be changed?
4. What are the **extended health care services** should be provided and undertaken by community pharmacist?

**Part III**

***Focus: Customers’ feedback towards community pharmacy practice-change***

1. What has been the **initial reaction from customers** when you have offered them advice and information? What feedback have you had from customer?
2. **How satisfied** do you think they have been with the advice?
3. Any **specific issues** that **customers usually raise with pharmacists** – have you been able to advise them on all queries so far?
4. Would more **training be helpful** in this area – any suggestions?
5. Any **customers returning for more advice** and / or for any feedback so far (improved customer wellbeing etc.)?

**Part IV**

***Focus: Future interventions***

1. Thoughts on offering **extended health care services for chronic conditions** as asthma, osteoporosis, hypertension, diabetes etc.? Any suggestions
2. If you have the **freedom**, how you offer an **intervention**? Explore any suggestions here – and listen for potential barriers etc.
3. In your opinion, what are the **barriers** that limit the health promotion activities in community pharmacy practice in Pakistan?

**Conclusion**

Any additional comments about community pharmacy practice in Pakistan?
